# Comparison of Complications of Chorionic Villus Sampling and Amniocentesis 

**Published:** 2012-03-20

**Authors:** Nahid Shahbazian, Mojgan Barati, Parvin Arian, Najmie Saadati

**Affiliations:** 1 Fetal Medicine Unit, Imam Khomeini Hospital, Jondishapor University of Ahvaz, Ahvaz, Iran; 2 Obstetrics and Gynecology Unit, Taleghani Hospital, Abadan Faculty of Medicial Sciences, Abadan, Iran

**Keywords:** Amniocentesis, Genetic Testing, Complications, Chorionic Villus Sampling

## Abstract

**Background:**

A significant number of pregnancies are associated with the cytogenetic abnormalities of the fetus. Amniocentesis and chorionic villus sampling (CVS) are procedures used for prenatal genetic diagnosis. In this study, we compare the safety and complications of mid-trimester amniocentesis and transabdominal CVS.

**Materials and Methods:**

This analytic cross-sectional study was performed in 308 patients from 2.11.2007 to 26.10.2009. We had 155 cases of amniocentesis, which we performed in weeks 15-23 of pregnancy; and 153 cases of CVS, which we performed during weeks 10-14 of pregnancy.

**Results:**

There were 2 cases (1.2%) of premature rupture of membrane (PROM) in amniocentesis which occurred 1 and 10 days after the procedure and caused pregnancy loss before 20 weeks. We had 1 case (0.7%) of abortion in CVS, which occurred 10 days after the procedure. Additionally, there was 1 case of amniotic fluid leakage (0.7%) in which, after admission to the hospital and observation, leakage was stopped and the pregnancy continued normally.

**Conclusion:**

In this study, we had more complications with amniocentesis cases than CVS. CVS is a procedure performed in the earlier stages of pregnancy and its complications are less than amniocentesis. We suggest CVS to be the procedure of choice for genetic diagnosis.

## Introduction

A significant number of pregnancies, especially in women with histories of infertility, are associated with cytogenetic abnormalities of the fetus (1). Methods such as chorionic villus sampling (CVS) and amniocentesis allow testing for chromosomal, genetic, and biochemical abnormalities (2). The traditional method of screening for Downs syndrome has been maternal age where amniocentesis or CVS is offered to women aged 35 years or more (3). The type of procedure selected depends on many factors, including indication, gestational age, and urgency of receiving the results (2).

Amniocentesis for genetic diagnosis is usually performed between 15 to 20 weeks (4, 5), and CVS is generally performed between 10 to 13 weeks.

Complications are infrequent in amniocentesis and include transient vaginal spotting or amniotic fluid leakage in 1-2% and chorioamnionitis in less than 0.1% (5). The incidence of amniotic fluid leakage or infection in CVS is less than 0.5% (6).

As a result of these findings, it is suggested that in the second trimester amniocentesis is safer than CVS. This conclusion has been supported by three randomized trials that have compared transcervical CVS and transabdominal CVS. Each trial recruited over 100 patients (7-9).

The safety of genetic amniocentesis has been addressed by several case-control studies and a randReceivedomized clinical trial (10-13).

The rupture of membranes, direct and indirect fetal injury, infection, and fetal loss are major complications associated with the procedures. Direct fetal needle injury during amniocentesis is rare with ultrasound guidance. Fetal loss of 0.5 percent or less has been reported by many investigators (4).

In a randomized trial reported by Philip and co-workers, the fetal loss rate that was associated with transab-dominal CVS before 20 weeks was 1.5% (14).

Maternal complications related to the procedure, such as amnionitis, are extremely rare, occurring in less than 1/1000 procedures (4, 5).

In this study we compared the safety and complications of CVS and amniocentesis. Awareness of complications is one of the most important factors in pregnancy, especially in patients with histories of infertility.

## Materials and Methods

This analytic cross-sectional study was performed in 308 patients from 2.11.2007 to 26.10.2009 in the Fetal Medicine Unit of Imam Khomeini Hospital in Jondishapor University. We had 155 cases of amniocentesis and 153 cases of CVS. Amniocentesis and CVS were performed in these patients for genetic analysis. Age, gravidity, parity, gestational age, and placental position were documented. Probable complications of premature rupture of membranes (PROM), abortion, intrauterine fetal death (IUFD), preterm delivery, infection, and leakage of amniotic fluid were analyzed.

Amniocentesis was performed between 15 and 20 weeks of gestational age; CVS was performed between 10 and 13 weeks (4, 5). An esaote mylab 20 transabdominal convex probe was used. The lower abdomen was prepped with antiseptic solution (alcohol).

Ultrasonographic guidance was used for amniocentesis to pass a 22-gauge spinal needle into the amniotic sac while avoiding the placenta, umbilical cord, and fetus. The first 2 cc of amniotic fluid was discarded in order to minimize the contamination with mother's blood or cells, then 20 ml of amniotic fluid was aspirated into a sterile syringe by gentle traction on thebarrel, then the syringe was removed. The fetal heart rate was assessed sonographically after the procedure. The amniocentesis sample was sent to the laboratory where cells were cultured, followed by chromosomal analysis (15).

For CVS ultrasonographic guidance was used to guide an l8-gauge spinal needle to the angle that allowed it to penetrate along the axis of the placenta. The stylet was removed, the medium - containing syringe mounted on the holder, and the holder was then attached to the hub of the needle. The needle tip was moved back and forth inside the placenta until an adequate sample was been aspirated. then the needle was removed. The medium was flashed on a tissue culture dish. All patients were followed until delivery. SPSS version 16.0 was used for analysis.

## Results

The maternal age varied between 16 and 45 years (mean: 31.1 ± 7.3 years) in amniocentesis cases. In CVS cases, maternal age range was between 17 and 41 years (mean: 25.4 ± 5.3 years). The mean gestational age was 17.3 weeks for amniocentesis cases and 12.1 weeks in CVS cases. The placental site was more anterior (52% in two procedures). Indications of both procedures are shown in table 1. Totally, 34 cases (11%) had abnormal results, of which details are shown in table 2.

We had 2 cases (1.2%) of PROM who underwent amniocentesis that occurred 1 and 10 days after the procedure, and caused pregnancy loss before 20 weeks. There was one case of IUFD that had undergone amniocentesis (0.6%), which occurred at 28 weeks; the fetus was hydropic. The cause of this fetal death was not related to the procedure. We had one case of preterm delivery in the amniocentesis group at 24 weeks gestation, which occurred 7 weeks after the procedure. There was 1 (0.7%) abortion in the CVS group, which occurred 10 days after the procedure. Also there was 1 case of leakage of amniotic fluid (0.7%) immediately after the procedure. The patient was admitted to the hospital for observation; the leakage was stopped and in this case, pregnancy continued normally.

In this study there were no cases of vaginal bleeding, chorioamnionitis or other maternal complications.

**Table 1:** Indications of amniocentesis and CVS


Indications	Amniocentesis		CVS	
	Number	%	Number	%

**High risk for major thalassemia**	37	23.87	129	84.31
**Advanced maternal age ≥35**	30	19.35	3	1.96
**Family history of Down syndrome**	22	14.19		
**Family history of genetic disorder + high Risk in triple screening test**	19	12.26	10	6.54
**High risk in triple screening test**	13	8.39		
**High risk for sickle cell**	6	3.87	9	5.88
**Previous births with fetal anomaly + high risk in first trimester screening test**	6	3.87	1	0.65
**Recurrent abortion history**	5	3.23	1	0.65
**High risk in triple test and advanced maternal age**	4	2.58		
**High risk in first trimester screening test**	2	1.29		
**Others**	11	7.09		


**Table 2:** Abnormal genetic results


Indications	Amniocentesis		CVS	
	Number	%	Number	%

**47XX+21**	4	2.58		
**45XO**	1	0.64		
**Major thalassemia**	4	2.58	24	15.69
**Sickle cell**			1	0.65


## Discussion

We had a 1.2% rate of PROM and abortion after amniocentesis, and 0.7% abortion after CVS. There was 1 case of IUFD in the amniocentesis (0.6%) group, which occurred at 28 weeks; the fetus was hydropic. We also had one case of preterm delivery in the amniocentesis group at 24 weeks. In our study, complications from CVS were less than complications of amniocentesis.

In a randomized trial performed by Philip et al. the rate of fetal loss associated with transabdominal CVS before 20 weeks was 1.5% (14). Alfirevic and collaborators found that midtrimester amniocentesis was safer than either transcervical CVS or early amniocentesis. They recommended transabdominal CVS if an early diagnosis was necessary (16). According to Philip et al. in their study, there was a pregnancy loss (less than 20 weeks gestation) for transabdominal CVS of 1.5% (14).

Fetal loss of 0.5% or less associated with amniocentesis has been reported by many investigators; further reductions have seemed impossible (17, 18).

Nanal and collaborators reported the total pregnancy loss rate for CVS to be 4.1% and a procedure–related loss rate of 0.23%. Their procedure–related loss rates for amniocentesis (0.7%) were calculated in a similar way (19).

CVS increased the risk of miscarriage from 0.6% (20) to 0.8% (21) more than mid-trimester amniocentesis. In the current study abnormal results have been detected in 34 out of 308 cases (11%).

## Conclusion

In this study we had a 1.2% abortion rate due to amniocentesis. Amniocentesis cases had higher complications than seen with CVS. CVS is a procedure that is performed earlier in pregnancy, and has less complications than amniocentesis. Thus, we suggest that CVS should be the procedure of choice for molecular genetic analysis.
